# Complete genome sequence of *Bradyrhizobium* sp. WCU1 isolated from lager beer

**DOI:** 10.1128/mra.01106-25

**Published:** 2026-03-16

**Authors:** André C. Velásquez, James D. Simon, Seán P. O’Connell

**Affiliations:** 1 Department of Biology, Western Carolina University 2739, Cullowhee, North Carolina, USA; University of Manitoba, Winnipeg, Canada

**Keywords:** *Bradyrhizobium*, genome, lager beer

## Abstract

In investigating potential flavor-altering microbiota in beer, we isolated a new bacterial species, *Bradyrhizobium* sp. WCU1, from a commercially available beer. We report its complete genome sequence, which includes genes for survival at low pH, growth on maltose, and for photosynthetic processes. The isolate does not contain nitrogen-fixation genes.

## ANNOUNCEMENT

Beers from multiple styles were tested to detect bacteria that could potentially alter beer flavor. Subsamples of 500 μL were spread-plated onto R2A media (1) and grown aerobically for 2 weeks at ~25°C in the dark. *Bradyrhizobium* sp. WCU1 was found in a Vienna-style lager purchased locally and originating from Mexico. Sanger sequencing of the 16S rRNA gene was performed by Azenta Life Sciences (South Plainfield, NJ) following their “Quick Lysis before PCR” using a proprietary primer set covering the V1–V9 hypervariable regions. Four *Bradyrhizobium* species matched 100% via BLAST alignment to the resulting 1,352 bp sequence (accessions NR_145861.1, NR_145860.1, NR_178802.1, and NR_178800.1). Based on these ambiguous results from 16S rRNA, we employed genome sequencing to identify the species and to assess which genes might be used *in situ* for WCU1’s growth and survival in beer.

For genome sequencing, WCU1 was grown in R2A broth media at 200 RPM and 30°C for 48–72 h. Cells were concentrated to ~6 × 10^9^ via centrifugation, washed in 1 mL sterile 1× PBS, and resuspended in 500 μL DNA/RNA Shield (Zymo Research, Inc.). DNA was extracted by Plasmidsaurus (Louisville, KY) using a Zymo Quick-DNA Fungal/Bacterial Miniprep Kit with RNase treatment, and then an amplification-free long-read library on unsheared DNA using Oxford Nanopore Technology (ONT) was constructed. The v14 library preparation chemistry with Rapid Barcoding Kit 96 V14 (SQK-RBK114.96) (2) was employed with minimal fragmentation of the input genomic DNA in a sequence-independent manner via tagmentation. The library was sequenced using R10.4.1 flow cells (2). Basecalling was completed using Dorado Super-Accurate Basecalling with default Q10 quality filtering. Reads were filtered using Filtlong v0.2.1 (default parameters), and the bottom 5% worst reads were removed. Miniasm v0.3 (3) was used for an initial assembly of 250-Mb reads from Filtlong and then re-downsampled to reach 100× coverage. The genome assembly was completed with Flye v2.9.1 (4) using parameters for high-quality ONT reads and then polished using Medaka v1.8.0. Contig analysis was performed with Bandage v0.8.1 (5), which showed that there was a circular chromosome and a plasmid; both were rotated to the optimal position using dnaapler. Genome completeness and contamination were checked with CheckM v1.2.2 (6). A total of 955 Mb were read encompassing 153,803 reads (N_50_ = 11,671) with a raw coverage of 124× and assembled coverage of 103×. The genome was annotated using RASTtk v1.073 in KBase (7, 8) using default parameters.

**Fig 1:**
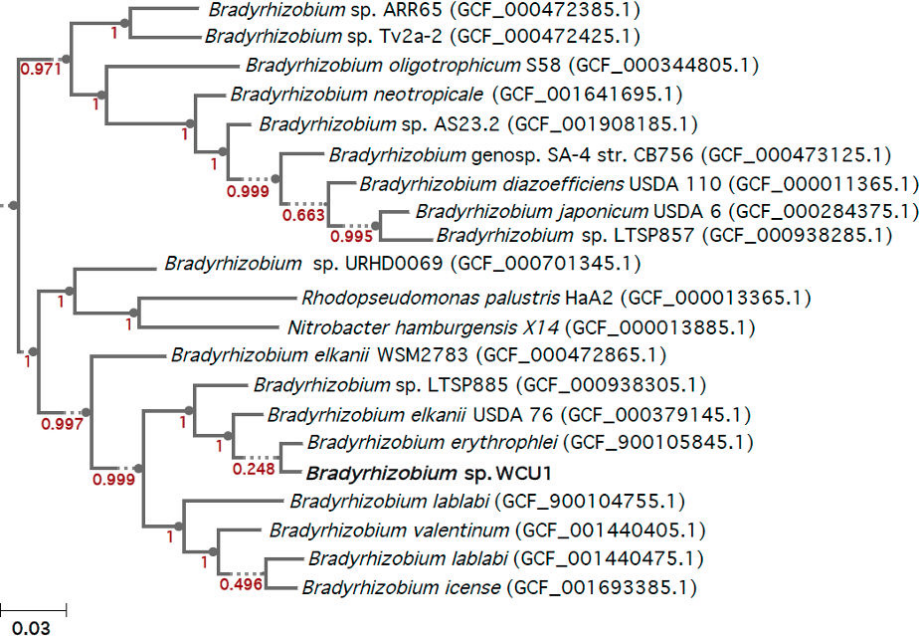
Maximum-likelihood phylogenetic tree for multiple sequence alignments (MSA) of 49 clusters of orthologous group (COG) gene families for *Bradyrhizobium* species including *Bradyrhizobium* sp. WCU1 (in bold) and two outgroup species from the Nitrobacteraceae. Numbers at branch nodes indicate bootstrap values. A tree was constructed using Insert Genome into Species Tree - v2.2.0 in KBase (https://doi.org/10.25982/217404.86/2589402).

WCU1 has a GC content of 64.5% and a genome of 7.7 Mb consisting of 7,421 genes. WCU1’s assignment in the *Bradyrhizobium* genus was confirmed on a maximum likelihood phylogenetic tree (Fig. 1). Genes for light-dependent reactions were found in a photosynthesis gene cluster (9) flanked by three transposases (Table 1). Genes favoring survival in acidic environments were present, while maltose—abundant in beer—degradation genes were also present. All type IV secretion components were found, mainly in the plasmid (10). Absence of nitrogenase and the genes for Nod factor production makes it unlikely that WCU1 can form a mutualistic interaction with plants (11).

**TABLE 1:** Genes of interest found in the genome of *Bradyrhizobium* sp. WCU1 highlighting functions that would allow the bacterium to survive in beer and/or could be the target for future study^*a*^

Function	Representative genes	Location of genes	Total number of genes
Photosynthesis			
Light reactions	*bch*(*x*), *crt*(*x*), *hem*(*x*), *puf*(*x*), and others	Chromosome	56
Calvin cycle	*acc*(*x*), *cbb*(*x*), *tktA_1*, *tktA_2*, and others	Chromosome	23
Denitrification	*nas*(*x*), *fix*(*x*), *nrt* (*x*), and others	Chromosome	20
Resistance to low pH	*exoF*, *exoR*, *actP_1*, and *actP_2*	Chromosome	4
Maltose utilization	*lamB*, *malK*, *malP*, and *malQ*	Chromosome	5
Type 4 secretion	*vir*(*x*), *chvG*, and *chvI*	Chromosome and plasmid	5 and 11
Flagellum biosynthesis	*fla*(*x*), *flg*(x), *fli*(x), *motA*, and *motB*	Chromosome and plasmid	46 and 2

^*a*^ *gene*(*x*) refers to pathways of the same function with three or more genes involved in that function; number of genes after the word "and" are those located in a plasmid.

## Data Availability

The raw sequencing reads have been deposited in the NCBI Sequence Read Archive (SRA) and can be found via the following link: https://www.ncbi.nlm.nih.gov/sra/PRJNA1307625. This Whole-Genome Shotgun (WGS) project has been deposited at GenBank under the accession JBQPHM000000000. The version described in this paper is version JBQPHM010000000. The KBase narrative including genome annotations using RASTtk (v1.073), Prokka (v1.14.5), and DRAM is available at https://doi.org/10.25982/217404.86/2589402.
